# 《血管性血友病诊断与治疗中国指南（2022年版）》解读

**DOI:** 10.3760/cma.j.issn.0253-2727.2022.01.003

**Published:** 2022-01

**Authors:** 杰 殷, 自强 余

血管性血友病（von Willebrand disease, VWD）是由于血管性血友病因子（von Willebrand factor, VWF）质或量的异常导致的临床以出血表现为主的遗传性疾病。近年来有关VWD高危人群筛选、临床诊断、治疗措施有了明显改变，为了推动国内VWD诊治的规范化发展，中华医学会血液学分会血栓与止血学组更新了《血管性血友病诊断与治疗中国指南（2022年版）》（简称指南）。在本文中，笔者对指南中的重要更新点作出详细说明。

一、正确使用出血评分工具

对于有出血症状的一般患者，指南推荐先使用国际血栓与止血学会出血评分工具（ISTH-BAT）进行筛查，确定是否需要进行进一步的实验室检查。指南中纳入的ISTH-BAT项目包括鼻出血、皮肤出血、轻微外伤出血、口腔出血、胃肠道出血、血尿、拔牙出血、手术后出血、月经出血、产后出血、肌肉出血、关节出血、中枢神经系统出血和其他出血症状，一共14项。每一项又根据出血程度，积分0～4分不等。ISTH-BAT是一个开放的出血评分工具，最后一项是其他出血症状，这意味着如果患者发生的出血事件不能归入前面13项，如咯血、肾上腺出血等，仍有相应的积分标准参照。对于反复多次出血的患者，每一项以最严重的出血症状计分，不同项目计分累加得到总积分[1]。男性总积分≥3分或女性总积分≥5分，即可判断为异常出血[1]。对于有多项出血表现和严重出血的患者，不容易漏诊。但是，对于病程短、出血次数少或出血症状轻的儿童，因ISTH-BAT积分不高，有漏诊VWD的风险，临床医生需谨慎评估[2]。因此，ISTH-BAT更适合成人出血患者的筛查。多项队列研究结果显示，ISTH-BAT诊断VWD的敏感性为75％（66％～83％），特异性为54％（29％～77％）[3]。VWD的诊断需要在ISTH-BAT筛选基础上，进一步行相关实验室检查。

二、VWD的实验室检查

为了有效和准确诊断与分型诊断VWD，指南纳入了更多的实验室检查。在VWD的诊断试验中，指南推荐血浆VWF血小板结合活性测定而非单一的VWF瑞斯托霉素辅因子活性（VWF∶RCo），用于反映VWF与血小板之间的结合活性。在VWD的分型诊断试验里，增加了去氨加压素（1-deamino-8-D-arginine vasopressin, DDAVP）试验、VWF前导肽（VWF propeptide, VWFpp）测定和VWF基因测序。

1. 血浆VWF血小板结合活性测定：指南推荐的血浆VWF血小板结合活性测定包括以下3种：①VWF与野生型小细胞膜糖蛋白Ⅰb（GPⅠb）结合测定（VWF∶GPⅠbR）；②VWF与GPⅠb突变体结合测定（VWF∶GPⅠbM）；③VWF瑞斯托霉素辅因子活性（VWF∶RCo）。

VWF∶GPⅠbR和VWF∶GPⅠbM由于变异系数小，检测阈值更低等优势，有替代VWF∶RCo的趋势[4]。但鉴于国内大部分单位还不能开展VWF∶GPⅠbR和VWF∶GPⅠbM检测，指南仍保留了VWF∶RCo。有些医疗中心采用VWF活性检测法[5]（检测试剂为识别VWF A1区上与GPⅠb结合肽段的单克隆抗体）替代VWF血小板结合活性测定。但基于指南的规范化，不推荐VWF活性检测法。

2. VWD的分型诊断试验：指南推荐部分VWD患者轻度出血时可使用DDAVP止血。推荐的前提是这部分患者需经过DDAVP试验证实有效[6]。DDAVP试验通过比较用药前和用药后1 h患者凝血因子Ⅷ（FⅧ）促凝活性（FⅧ∶C）和VWF水平的变化，初步判断是否有效。用药后4 h再次检测患者FⅧ∶C和VWF水平，判断患者是否存在VWF代谢过快、半衰期缩短。例如1C型患者，用药后1 h VWF水平升高达到有效标准，但用药后4 h VWF水平下降幅度超过30％，提示用药后临床上达不到稳定止血效果，因此这类患者不推荐使用DDAVP治疗。DDAVP试验用药和操作并不复杂，笔者单位在门诊可实施，但禁用于2岁以下的婴幼儿、妊娠妇女、癫痫及有活动性心脑血管疾病的老年患者 [6]。

VWFpp和成熟VWF等比例地从细胞内分泌出来进入血循环。VWFpp在体内的半衰期是2 h，成熟VWF的半衰期是8～12 h。所以，VWFpp和VWF抗原（VWF∶Ag）之间的比值可以反映体内VWF合成、分泌及清除的状况。由于VWFpp不含ABO血型抗原，所以VWFpp的清除不受血型影响[7]。VWFpp与VWF∶Ag在体内代谢途径不同，因此VWFpp/VWF∶Ag增加提示VWF清除增快。VWFpp和VWF∶Ag均采用ELISA方法检测，正常血浆中含有1 U的VWFpp和1 U的VWF∶Ag，两者比值为1[8]。在1C型VWD和获得性von Willebrand综合征中，VWFpp/VWF∶Ag明显增高[9]。

目前VWF基因测序越来越广泛应用于VWD的诊断。VWF某些特定的外显子突变与VWD分型密切相关，对于VWD的鉴别诊断也具有重要价值。但是，不可否认的是仍有部分VWD患者找不到VWF的基因突变，这些患者可能还存在VWF细胞内转运、分泌、调控、代谢等其他因素的异常[10]，因此不能仅依据VWF基因检测结果正常即排除VWD。此外，如患者没有明显的出血表现或VWF水平的异常，即使检测到VWF基因异常，临床医生还需根据患者既往是否有出血病史和出血性家族史，慎重鉴别是VWF的基因多态性还是致病性的VWF基因突变，诊断或排除VWD。

三、VWD的诊断

本版指南新增了VWD的诊断流程图。由于DDAVP试验的引入，首次提出了1C型VWD的诊断。为了避免漏诊，指南明确将VWF血小板结合活性/VWF∶Ag比值<0.7作为2型VWD的诊断标准[4]，而非之前的0.5～0.7。3型VWD患者的VWF水平通常低于检测阈值，VWF多聚体缺如[4],[11]。因国内大部分医院无法常规开展VWF多聚体检测，所以需要明确界定VWF∶Ag和VWF∶RCo阈值。兼顾VWF∶RCo检测下限的准确性，及VWF多聚体缺如的3型VWD患者的VWF水平，指南最终将3型VWD的VWF抗原和VWF∶RCo阈值划为小于3％。在2A和2B型VWD的鉴别中，更推荐采用基因测序而非低剂量瑞斯托霉素诱导的血小板聚集试验，原因在于后者需要患者新鲜血小板进行检测，临床普及性不及VWF基因测序[4]，并非低剂量瑞斯托霉素诱导的血小板聚集试验诊断2B型VWD准确性低。

四、VWD的预防及治疗

中重度出血或拟行手术的VWD患者，需替代治疗。目前国内替代治疗可选择人血源性FⅧ制剂、血浆和冷沉淀。近期人血源性VWF制剂将开展临床试验。剂量标定以制剂的VWF∶RCo为准，每公斤体重1 U的VWF∶RCo平均使血浆VWF∶RCo提高2 U/dl。针对不同程度出血和拟行手术类型，不同版本国内外指南推荐首次用药、维持用药剂量和疗程均不相同[11]–[13]，因此本指南列出了替代治疗的推荐范围。具体到每例VWD患者，则应兼顾用药后出血情况、VWF和FⅧ水平以及替代治疗制剂的选择进行个体化调整[14]。重组VWF制剂未来也将在国内上市，需注意的是，与血源性VWF制剂不同，重组VWF制剂不含有FⅧ。在严重出血或手术时，首次应用为了能迅速提升患者体内的FⅧ水平而达到止血效果，应同时补充1剂FⅧ，随后可单用重组VWF制剂维持[15]。

指南针对女性VWD患者的月经过多、出血性卵巢囊肿、妊娠和分娩等问题均提出了明确的治疗措施。正常女性妊娠期VWF和FⅧ∶C水平增加2～3倍，1型VWD孕妇也有类似表现。因此，在妊娠后期应监测VWF水平，如能达到正常范围，分娩时可不予以干预。分娩后患者的VWF水平迅速下降，需要严密监测。研究显示，即便分娩时VWF水平处于正常范围，仍有22％的VWD患者因产后异常出血需要输血[16]。抗纤溶药物氨甲环酸在女性VWD治疗中占重要地位，无论是月经出血、出血性卵巢囊肿还是预防产后出血均可使用，可单药应用，也可联合替代治疗。值得一提的是，产妇服用氨甲环酸，母乳中虽然可以检测到氨甲环酸，但仅有血液浓度的百分之一[17]，因此相对安全，可用于哺乳期VWD患者。VWD产妇除了需预防产后大出血，同样也面临产后血栓的风险。对于合并血栓高风险的患者，可用弹力袜预防，必要时也可考虑低分子肝素[6]。

指南将手术分为大手术和小手术两种类型并加以定义。对于拟行大手术的VWD患者，在替代治疗的同时，需检测FⅧ和VWF水平，两者均需达到0.50 IU/ml并至少维持到术后3 d。仅FⅧ或VWF水平达标，术中及术后仍有出血的风险[6]。VWD患者合并心血管疾病或血栓性疾病而需要抗血小板或抗凝治疗时，建议行抗血小板或抗凝治疗，如治疗后合并出血，可加用预防治疗。

当然，除了指南中推荐的VWD治疗制剂外，新的VWD治疗策略也层出不穷。VWD基因治疗、VWF-FⅧ融合蛋白、长半衰期VWF纳米制剂等不仅给VWD患者带来新的希望，也为临床医生提供了更多的治疗选择[18]。

随着血源性/重组VWF制剂的上市和推广，预防治疗也变得可及，反复发生严重出血的VWD患者将获益于预防治疗。关于VWD的预防治疗还存在诸多问题，诸如哪一类型VWD患者获益更多、长期预防是否会导致VWF抑制物产生、预防治疗的最佳剂量和频率、选择阶段性预防治疗还是终身预防治疗等。以上问题需要开展更多的临床试验予以解答。
